# Editorial: Glial cells as an emerging therapeutic target in the pathobiology of central nervous system disorders: friend or foe?

**DOI:** 10.3389/fncel.2023.1191743

**Published:** 2023-04-25

**Authors:** Nima Sanadgol

**Affiliations:** Institute of Neuroanatomy, RWTH University Hospital Aachen, Aachen, Germany

**Keywords:** astrogliosis, microgliosis, oligodendrocytes, ependymal cells, neurodegeneration, neuroinflammation

Central nervous system (CNS) disorders and related diseases may result from the direct or indirect role of immune cells in the nervous system and are clinical features of numerous neuropsychological and neurodegenerative disorders (Passaro et al., 2021). In addition to serving as a pathological marker, the brain's innate immune system dysregulation is thought to play a crucial role in the development of neuropathies, finally resulting in the secretion of inflammatory mediators and neurotoxins from activated resident macrophages (microglia) and astrocytes that promote neuronal damage and death (Kölliker-Frers et al., 2021). Recent data suggest that the role of glial-derived inflammation in neuropathies must be fully elucidated, since pro-inflammatory agents, occur widely, particularly in the acute phases, and are also linked to the activation of neurogenesis and direction of neural stem cells to the lesion site (Kizil et al., 2015; Sanadgol et al., 2018; Houshmand et al., 2019). So, it is supposed that anti-inflammatory treatments sometimes hinder this navigation and have more effects on the management of conditions rather than recovery of functional and mental impairments (Ebrahimy et al., 2022; Gilbert et al., 2022). Overall, the exact role of glial-derived inflammatory/anti-inflammatory mediators on neural regeneration has been controversial. To provide an outline of this topic, we have selected five original research and review articles that explain the role of neuroinflammation (a process whereby the brain will encounter an inflammatory challenge) and its related pathogenic processes in a variety of neuropathies.

In one article, Miah et al. reviewed the effect of cell composition of olfactory cell transplantation on the therapeutic outcomes in spinal cord injury (SCI). Regeneration of olfactory sensory neurons is thought to be extremely dependent on olfactory ensheathing cells (OECs, specialized glial cells). OECs exhibit unique neurotrophic properties and play a vital role in supporting and guiding newly formed olfactory sensory axons from their origin to establish synaptic connections with their targets in the normally non-permissive CNS environment of the olfactory bulb to facilitate odor detection. This review provides new insights into olfactory tissue culture and transplantation on therapeutic outcomes in SCI.

In an original research report, Wang X. et al. demonstrate that β-1,4 Galactosyltransferase V (β-1,4-GalT V) is involved in microglial function. β-1,4-GalT V is a galactosyltransferase mainly located in Golgi, and participates in the regulation of protein glycosylated modification. They found that β-1,4-GalT V affects the expression level of tumor necrosis factor receptor (TNFR)2 instead of TNFR1. They demonstrated that β-1,4-GalT V expressed in microglia and regulated microglial migration, proliferation, and release of their related inflammatory factors. They concluded that β-1,4-GalT V is expressed in microglia and has an impact on microglia function and represents a promising therapeutic target during traumatic brain injury (TBI) and other neuroinflammation-mediated diseases.

A brief research report by Wang Y. et al. highlights that suppressing neuroinflammation during the early post-surgical phase can limit long-term deficits in both behavioral and neurogenic outcomes after rat model of cardiopulmonary bypass (CPB). Post-operative cognitive dysfunction (POCD) can be a serious surgical complication, and patients undergoing cardiac procedures are at particular risk for POCD. Treatment with minocycline reduced neuroinflammation and microglia/macrophage reactivity after 6 months post-CPB in the dentate gyrus of the hippocampus. In addition, the CPB-induced reduction in adult neurogenesis was attenuated in the minocycline-treated animals. Such an approach would greatly facilitate the development of novel intervention strategies in order to decrease the risk of POCD in patients undergoing cardiac procedures.

In another original research report, Rosato et al. proposed a novel *in vitro* model based on Müller cells, a type of retinal glial cells, that may be useful for further investigation of potential antioxidant and anti-inflammatory candidates for the prevention and/or treatment of diabetic retinopathy (DR). DR is a common complication of diabetes mellitus and is the major cause of vision loss in the working-age population. Although DR is considered a microvascular disease, recently an increasing body of evidence suggests that neurodegeneration is an early event that occurs even before the manifestation of vasculopathy. The authors found that hyperglycemic-like condition is well-tolerated by Müller cell line rMC-1 but makes them more susceptible to a pro-inflammatory environment. More specifically, rMC-1 cells exposed to high glucose decrease their ability to counteract oxidative stress, with consequent toxic effects. Although limited to an *in vitro* model of Müller cells, their experiments provide new insights into the relationship between these cells and the typical DR environment.

In another original research report, Arik et al. investigated whether recombinant human erythropoietin (rhEPO) could prevent or significantly decrease microglial reactivity and the subsequent induction of inflammasomes after ischemia. Erythropoietin is a glycoprotein hormone, naturally produced by the peritubular cells of the kidney, that stimulates red blood cell production.

The authors found that rhEPO administration in human microglial clone 3 (HMC-3) cells mitigated OGD/R-induced oxidative stress and cell death and enhanced metabolic activity, migration, and phagocytosis of these cells. Additionally, rhEPO attenuated post-ischemic activation and regulation of the inflammasomes (NLRP1, and NLRC4) as well as its downstream effectors. They provide evidence that EPO-conveyed anti-inflammatory actions might be mediated *via* the regulation of the inflammasomes.

The selected articles will provide readers with an understanding of molecular mechanisms governing glial-related neuroinflammation and neuropathy, in addition to demonstrating how anti-therapeutic strategies might help to limit the pathogenesis of certain diseases.

## Author contributions

The author confirms being the sole contributor of this work and has approved it for publication.
